# Reconciling pillars of transient gene expression: From DNA prep via media, reagent and cell line development to holistic process optimization

**DOI:** 10.1186/1753-6561-9-S9-P18

**Published:** 2015-12-14

**Authors:** Sebastian Püngel, Miklos Veiczi, Tim F Beckmann, Vanessa Vater, Penélope V Soto, Renée Ermerling, Derek Levison, Christoph Heinrich, Tim Welsink

**Affiliations:** 1 InVivo BioTech Services GmbH, 16761 Hennigsdorf, Germany; 2 emp Biotech GmbH, 13125 Berlin, Germany; 3 Xell AG, 33613 Bielefeld, Germany

## Background

Transient gene expression (TGE) is a multi-parametric process which is built upon four essential influencing pillars: First of all, it is imperative to have an easy to transfect cell line which allows high product titers and is cultivated in suspension [1]. Secondly, only a few of the commercially available cell culture media allow / support transient transfection and production. Thus, it is highly recommended to select the appropriate medium to grow and transfect the favored cell line [2]. Thirdly, the quality, source and backbone of the plasmid DNA which contains the genetic information for the protein of interest has a major impact [3]. Last but not least, to introduce plasmid DNA into mammalian cells, selection of a suited transfection reagent plays an important role for high-yielding TGE processes.

In this work, we summarize our recent development of a novel TGE system for efficient transient transfection and expression in HEK cells. In cooperation with emp Biotech, InVivo BioTech Services developed a transfection reagent with very low cytotoxicity. A culture medium that can be used for transfection and production was designed in collaboration with Xell AG. The establishment of a TGE optimized HEK cell line (HEK-INV) and a method for large scale plasmid preparation of a corresponding vector complete the optimized production platform. It is easy applicable, scalable and supports large scale transfection for the production of gram quantities of IgG within a few days.

## Materials and methods

Mammalian cells were cultivated and transfected in Xellvivo TM medium (Cat. No. 861-0001, Xell AG) under conditions of 37 °C, 5 % CO2 and 185 rpm agitation speed at 50 mm orbital diameter. For screening approaches 5x10E6 cells/mL were transfected with 2 pg DNA/cell and INVect transfection reagent (Cat. No. FK-0101-M001.0-001, emp Biotech GmbH) with INVect to DNA ratio of 6:1 (w/w) or 25 kDa L-PEI with PEI to DNA ration of 2:1 (w/w) in 8 mL culture volume in 50 mL bioreactor tubes. Expression of IgG1 was performed in 30 mL culture volume in 125 mL shake flasks or in 150 mL culture volume in 500 mL shake flasks respectively. Yields were quantified by proteinA affinity chromatography. Directed evolution was performed referring to Majors et al., 2009 [4]. In detail, an iterative process of evolution rounds followed by analysis of favorable attributes, cell selection and recovery was implemented. The corresponding flow cytometry analysis was performed using a Bio-Rad S3 cell sorter.

Several E.coli strains and media were screened for high productivity, high quality and flexibility for DNA preparation in comparison to commercial kits in mini-preparation scale. A purification process was implemented using a reusable and scalable anion exchanger. For large scale plasmid preparation, 6 L suspension was lysed, clarified and purified using an Äktaprime chromatography system. DoE was used for TGE process optimization after combining all developed elements.

## Results

**Figure 1:**
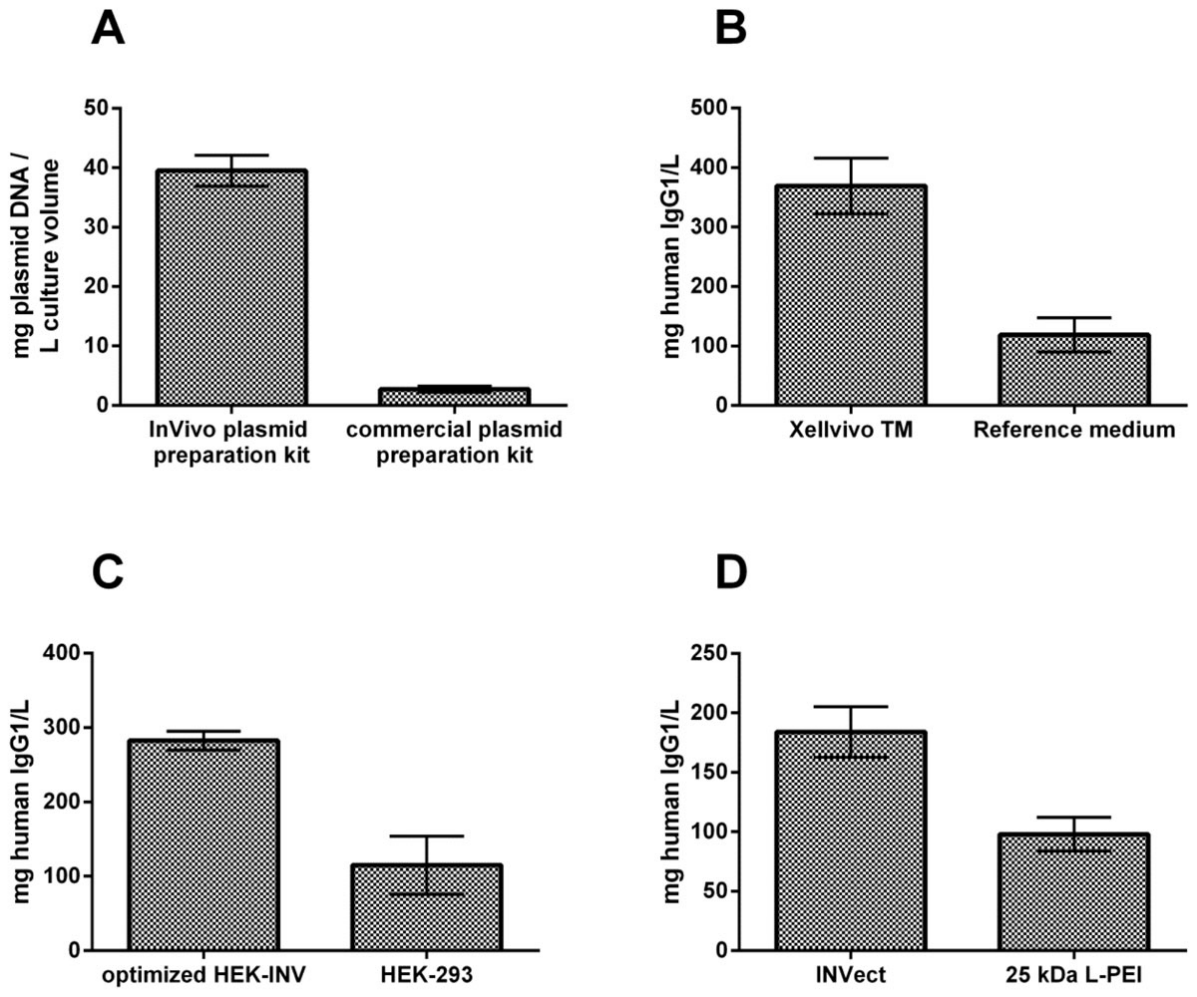
**Overview of performed optimizations of TGE parameters**. (a) Yield of plasmid preparation for transient transfection. (b) Antibody quantities (7 day post transfection) expressed by HEK-293 using indicated media. (c) Antibody quantities (7 day post transfection) expressed by HEK-293 and proprietary HEK-INV. (d) Antibody quantities (7 day post transfection) expressed by HEK-293 using indicated transfection reagent.

At least two plasmids were compared using different E. coli strains cultivated in three different media in regards to yield and quality aspects. Quality attributes such as the amount of supercoiled monomeric DNA highly depended on the host strains, whereas absolute yield was found to be a function of the cultivation medium. Additionally, a purification process was implemented using an anion exchanger. Four independent runs resulted on average in 40 mg plasmid DNA/L culture volume (Figure 1A). However, somewhat unexpectedly, endotoxin levels up to 50,000 EU/mL do not appear to influence viability, transfection efficiency nor do they have any marked effect on productivity. Starting from a basal medium for mammalian culture, a novel medium which supports both transient transfection and high titer transient gene expression was generated in cooperation with Xell AG. Improvements were achieved by stepwise screening and optimization of media components with regards to higher cell growth, transfection efficiency and productivity. The final medium formulation led to a 4-fold increase in IgG1 productivity (Figure 1B). To generate an optimized host cell line for TGE processes, we utilized directed evolution which resulted in a 3-fold increase in IgG1 productivity in comparison to the parental host cell line (Figure 1C). Commonly used transfection reagents such as polyethyleneimine (PEI) are quite cytotoxic when used in high concentrations. As a consequence, the maximum transfectable cell density is limited, since about 0.5 pg DNA/cell are required during transfection. As a result, similar levels of production required much larger cell culture vessels using 25 kDa L-PEI. The newly developed INVect is a transfection reagent which demonstrates low cell toxicity for transient transfection of mammalian cells and leads to extremely high transfection efficiencies up to 90 %, 24 h post transfection. Finally, the use of INVect for transfection at TGE conditions resulted in high levels of protein expression with 2-fold increase in IgG productivity compared to 25 kDa linear PEI (Figure 1D).

The combination of all developed aspects allows two process strategies. On one hand, the low toxicity of INVect and the performance of the new media system allow transfection and cultivation at high cell densities (about 1-4x10E7 cells/mL) achieving antibody titers up to 850 mg/L (Table 1). On the other hand, it was possible to establish the first high-yielding pseudo-perfusion TGE production process at high densities (about 4x10E7 cells/mL) which enables maximum space time yields up to 200 mg IgG per reactor volume and day.

**Table 1:** Transient expression of a monoclonal antibody in independent experiments with differing setups and scales.

	Pseudo-perfusion	Batch	Fed-Batch
Viable cell density	40 x10^6 ^cell/mL	5 x10^6 ^cell/mL	20 x10^6 ^cell/mL
Volume [mL]	10	30-3000	30-300
Duration [days]	10	7	6
Yield [mg/L]	240*	400	850

* final antibody concentration in perfundate

## Conclusions

In conclusion, InVivo's TGE system includes an advanced cell line and vector system, a novel transfection reagent as well as a unique and customized media, all synergistically optimized for highly efficient production of recombinant proteins. Applications of this streamlined process are in early development and lead identification as well as gram-scale production for pre-clinical trials. Furthermore, a pseudo-perfusion TGE process was developed for the production of toxic or labile products such as enzymes or vaccines. Lastly, a simplified procedure of the production process using a customized concentrated feed supplement was implemented which resulted in antibody titers up to 850 mg/L. Subsequent screenings of transfection enhancers show promising results and indicate potential for further improvements.
